# Case of a Woman Who, after Having Been Gored in the Abdomen by an Ox in the Sixth Month of Pregnancy, Underwent the Cæsarean Operation

**Published:** 1790

**Authors:** Frederick Augustus Fritse

**Affiliations:** Physician at Dillenburgh


VI. *Caſe of a Woman who, after having been gored in the Abdomen by an Ox in the ſixth Month of Pregnancy, underwent the Cæſarean Operation. By* Frederick Auguſtus Fritſe, *M. D. Phyſician at Dillenburgh.*—Vide *Vermiſchte Chirurgiſche Schriften*; i. e. *Miſcellaneous Chirurgical Eſſays, collected and publiſhed by* J. L. Schmucker, *Surgeon General of the Pruſſian Army. Volume the Third.* 8vo. Berlin.

THE ſubject of this very curious and extraordinary caſe was a poor woman, named Schulers, at Offdillen, in the principality of Dillenburgh, of a delicate habit of body, but in other reſpects healthy, who had already borne ſeveral children.

About two o'clock in the afternoon, of the 20th of October, 1779, being then in the ſixth month of her pregnancy, ſhe was gored by an ox in the lower belly. The horn of the animal penetrated the right hypogaſtric region, at the diſtance of about three inches from the linea alba, and wounded the body of the uterus.

Her huſband, who ſaw her in this dreadful ſituation, holding by the other horn, flew to her affiftance, and, in difentangling her from the animal, unfortunately made a frefh wound, two inches in length, with the horn that was ftill fticking in the abdomen. This fecond wound, which was exactly in the direction of the linea alba, was attended with a laceration of the parts, and with a lofs of fubftance, fo as to form one common wound with the firft.

A confiderable hæmorrhage enfued; and, on examination, the right arm of the fœtus was found protruding through the wound.

Dr. Fritfe, whofe affiftance was called for, but who did not get to her till ten hours after the accident, found her ftrength, he tells us, lefs funk than, confidering the nature of the cafe, might have been expected; the hæmorrhage, which at firft was confiderable, had almoft entirely ceafed; her pulfe had ftill a certain degree of fulnefs, and her breathing was not materially affected.

After having duly confidered all the circumftances of the cafe, being convinced of the neceffity of having recourfe to the Cæfarean operation, as the only probable means of preferving the life of the patient, he communicated this opinion to her in a proper manner, and fhe with great firmnefs determined to fubmit to it.

As it was now paſt midnight, and no ſymptoms were preſent that indicated a neceſſity of performing it immediately, he thought it better to defer the operation till the morning than to attempt to perform it by candle light. He, therefore, employed himſelf during the remainder of the night in directing the patient's chamber to be properly ventilated, in adminiſtering to her ſuch remedies as her caſe ſeemed to require, and in ſecuring the wound, as much as poſſible, from the external air.

At ſeven o'clock the next morning, after having paſſed a very reſtleſs night, ſhe voided her urine without any conſiderable pain; but as ſhe had had no ſtool during the laſt four-and-twenty hours, it was thought right, previouſly to the operation, to adminiſter a clyſter, but it failed to produce the deſired effect.

The patient being now placed in a poſture convenient for the operation, and properly held by four aſſiſtants, the operator placed himſelf on her left ſide, that he might have the free uſe of his right hand.

He firſt introduced his left fore finger into that part of the wound where the horn of the ox had penetrated into the cavity of the abdomen, and which, as hath been already ob-

ſerved, was three inches diſtant from the linea alba, and about as far from the abdominal ring.

After having ſufficiently enlarged the wound with the point of his ſcalpel to admit another finger, he introduced into it his middle finger; and then, cutting through the right muſculus rectus abdominis and the peritonæum, enlarged the wound three inches, in a *ſtraight line* upwards towards the navel; after which, taking hold of the knife with his left hand, he carefully cut downwards, to the extent of an inch, toward the right abdominal ring.

The opening in the abdomen being now ſufficiently large to allow of the extraction of the fœtus, he began to examine the ſtate of the uterus, and, by degrees, ſucceeded in introducing his right fore finger into its cavity through the wound which had ſo ſtrongly contracted about the arm of the fœtus, and by this means was enabled to aſcertain the ſituation of the child, and likewiſe to ſatisfy himſelf that the placenta was attached to the right ſide of the uterus, and not to the fundus. Being thus convinced that he had nothing to fear from cutting through the fundus uteri, he introduced his fore and middle fingers between the uterus and the body of the child, and then cut through the body of the uterus and its fundus to the extent of four inches.

The fœtus was now brought out through the wound, though not without ſome difficulty, and much pain to the patient; as was alſo the placenta, which was found to be firmly attached. As ſoon as all this was accompliſhed, the operator again introduced his hand into the uterus to clear it of coagulated blood.

The head of the child was no ſooner brought out through the external wound than the colon began to protrude, but was kept up by one of the aſſiſtants till the operator had done with the uterus, and then he reduced it in the uſual manner.

The integuments of the abdomen being now brought together by means of the interrupted ſuture, as it is called, ſuitable dreſſings were applied to the wound, and ſecured by a bandage, three inches broad and eighteen ells in length.

The whole of the operation, we are told, including the application of the bandage, did not take up more than a quarter of an hour; and only twice, in the courſe of it, did the patient complain of pain, namely, when the head of of the child was brought out through the wound of the uterus, and, afterwards, on the placenta's being ſeparated. The loſs of blood, during the operation, is ſaid not to have exceeded ſeven ounces.

The patient was now carefully placed, in an eaſy poſture, in bed; a little wine was given her to drink; and ſhe was allowed to take freely of chicken broth.

About noon a diſcharge of blood began to penetrate a little through the dreſſings and bandage, but without any appearance of conſiderable hæmorrhage; and even at this early period the lochia were beginning to flow.

The only complaint the patient made was of pains like after-pains, which, from her manner of living previouſly to the accident, it was thought might be occaſioned in ſome meaſure, or at leaſt increaſed, by impurities in the primæ viæ. Another clyſter was, therefore, directed to be adminiſtered, but this, like the former one, came away without producing any evacuation of fæces. The bandage was moiſtened with vinegar and ſpirit of wine, diluted with water; and for the purpoſe of allaying thirſt ſhe drank water acidulated with vinegar, and rendered palatable by the addition of ſyrup of raſpberries.

raſpberries. She alſo took, from time to time, ſmall doſes of nitre.

At two o'clock in the afternoon the pains were not at all diminiſhed, and ſhe began to be troubled with nauſea and eructations. Another clyſter was therefore injected, but without the deſired effect. At five o'clock ſhe complained of pain about her cheſt, and of an increaſe of the nauſea, and about two hours afterwards, after vomiting bile, ſhe found herſelf relieved, a gentle perſpiration took place, and ſhe ſlept quietly during a conſiderable part of the night.

The day following, being the third after the accident, the bandage was ſuffered to remain undiſturbed, for fear of occaſioning a freſh hæmorrhage, and the ſame plan of treatment was purſued as on the preceding day.

In the forenoon of this day ſhe complained again of pain of the abdomen, and of a ſenſation as if this part was ſwelled; but, on examination, no enlargement or hardneſs of the belly could be perceived. She was frequently troubled with acid eructations, and her pulſe was now much quickened.

As ſhe had as yet had no ſtool ſince the operation, another clyſter was adminiſtered, conſiſting of two parts water, one part vinegar, half half an ounce of common ſalt, and a little linſeed oil. This very ſoon had the deſired effect, to the great relief of the patient; a copious perſpiration enſued; her breathing was free and natural; and ſhe got ſome more ſleep.

About five o'clock in the afternoon the pains of the belly and eructations again returning, ſhe drank an infuſion of chamomile flowers, which ſhe thought afforded her relief; but the moſt certain remedy, it is obſerved, for theſe complaints was found to be the clyſter of water and vinegar: for, in the courſe of the treatment, ſhe had frequent returns of the pains of the abdomen, acid eructation, nauſea, and even vomiting, but theſe ſymptoms never failed ſpeedily to give way to the uſe of the ſort of clyſter juſt now mentioned.

On the fourth day ſhe was reported to have ſlept well during the night, but at eight o'clock in the morning was found to have a conſiderable degree of fever.

The bandage was now removed for the firſt time. The lips of the inciſed wound were in contact with each other, and ſlightly inflamed; thoſe of the lacerated wound were an inch aſunder, and greatly inflamed.

 A decoc-

A decoction of myrrh in barley water, with the addition of a little honey of roses, was now injected into the cavity of the abdomen, (a mode of practice which will probably not accord with the ideas of the English surgical reader) and the same kind of bandage was applied as before.

Suitable remedies were given to moderate the quickness of pulse, thirst, and other symptoms of fever. The patient's common drink was water properly acidulated, and she also took chamomile tea, and a decoction of bark, with Hoffmann's anodyne liquor.

On the fifth day, the patient having passed a good night, and slept well, found herself much better. The hardness and quickness of pulse were much diminished; the uneasy sensation she had complained of in the abdomen had subsided; she had had a natural stool, had made water, and was now in a gentle perspiration.

The bandage and dressings were again removed; the injection into the cavity of the abdomen was repeated; and the abdomen was gently embrocated with a mixture of vinegar and oil of chamomile.

On the sixth day the patient had three stools. The state of the pulse indicated an increase of fever; fever; and the night following ſhe was reſtleſs, and troubled with a frequent cough, which, ſhe ſaid, occaſioned, every time ſhe coughed, a ſmarting pain in the wound.

On removing the dreſſings this day, it was obſerved that the ligature of the inciſed wound had ſo cut through the ſkin, that the lips of the wound were ſeparated from each other at the outer ſurface, but that at the bottom of the wound an union of parts had taken place. As the ligatures, therefore, now ſeemed to be no longer of any uſe, they were removed, and the lips of the wound were kept together by means of ſlips of ſticking plaſter.

On the eighth day the patient had two ſtools; the cough was leſs troubleſome, and the wound was beginning to diſcharge a good pus.

On the fifteenth day, as the patient ſtill complained at times of the cough, a few drops of laudanum were occaſionally added to her medicines.

At this period the wounds appeared to be in a good condition, and the patient was in every other reſpect in a comfortable ſtate: ſhe ſlept well, the ſymptoms of fever had entirely ſubſided, her evacuations by ſtool and urine were natural, and her appetite for food had returned.

On the ſeventeenth day a ſlight excoriation of the ſkin around the wound, occaſioned by the diſcharge, made it neceſſary to recommend a ſuitable topical application, which ſoon produced the deſired effect.

On the twenty-fourth day a conſiderable diſcharge of matter was obſerved on the bandages, and traced to a ſmall abſceſs between the integuments and the muſculus rectus abdominis, which was ſoon healed by means of gentle compreſſes.

On the thirty-fourth day, the wound being almoſt completely healed, a proper bandage was applied to prevent a hernia; after which the patient, for the firſt time after the operation, walked about her chamber.

On the 21ſt of December ſhe was ſufficiently recovered to return to her uſual occupations; and towards the latter end of February, about four months after the operation, ſhe came on foot to Dillenburgh, a fatiguing journey of two (German) miles.

*Sequel of the Caſe.*

During the ſpring of the year 1780 the patient continued in perfect health, and engaged in the moſt laborious occupations, without experiencing periencing any inconvenience from the operation.

In the month of Auguſt of that year ſhe became again pregnant, and, during the firſt four months of her pregnancy, experienced no other ſymptoms than thoſe peculiar to women in general in that ſtate. But at the end of that period the cicatrix of the lacerated wound began to grow thinner than the reſt of the integuments, and to protrude more and more, ſo that in the month of February, 1781, the cicatrix was become extremely thin, and the projection of the abdomen at that part, towards the right ſide, was very conſiderable.

The abdomen was ſupported with all poſſible care by means of bandages paſſed round the pendulous belly and over the ſhoulders till the 28th of April, when the patient was delivered, after a very eaſy labour, of a dead child.

She was immediately put to bed with the placenta ſtill in utero. In this ſtate ſhe was found by our author ſix hours after the delivery of the child. She complained hardly of any pain, and the lochia flowed very ſparingly; but her thirſt was exceſſive, and the powers of life were greatly greatly reduced; ſhe had a quick ſucceſſion of fainting fits, and died in leſs than an hour after his arrival.

The following were the appearances that occurred on examining the body after death:

The abdomen was obſerved to be conſiderably diſtended.

The cicatrix of the wound made by the Cæſarean operation was found to be perfectly firm; while, on the other hand, that of the lacerated wound was extremely thin and diſtended.

Upon opening the cavity of the abdomen there were found in it twelve pounds of extravaſated blood, partly in a fluid and partly in a coagulated ſtate.

The peritonæum adhered firmly to both the cicatrices, and the omentum was alſo found to have contracted ſome adheſions to the peritonæum at thoſe places.

On the right ſide of the abdomen, anteriorly, a portion of the inteſtinum cœcum was found adhering pretty firmly to that part of the peritonæum which is reflected over the uterus.

The veins of the uterus, and particularly of the the right ovary, were ſo much enlarged, that the natural figure of the latter could with difficulty be diſtinguiſhed.

A coagulum of blood, lodged between the right ovary and the right lateral portion of the fundus and body of the uterus, had diſtended the peritonæum reflected over thoſe parts ſo as to form a ſac, with an aperture through which the blood had made its way into the cavity of the abdomen. Upon carefully cleanſing this ſac of its contents it was found that ſeveral of the enlarged veins of the ſubſtance of the uterus opened into it.

The place where the inciſion had been formerly made in the ſubſtance of the uterus by the Cæſarean operation was ſo perfectly cicatriſed, that it could hardly, with certainty, be diſtinguiſhed; and the portion of the uterus through which the horn had penetrated, and where there had been a loſs of ſubſtance, was likewiſe ſo completely healed, that nothing but the thinneſs of the uterus at that part ſerved to point it out.

The placenta was ſtill ſo firmly adherent to the uterus, that it required much pains to ſeparate it without injury to the latter. After the removal of the placenta, the uterus was taken out and dried, and then filled with water, not a drop of which made its escape.

All the other abdominal viscera were in a natural state.

That the effusion of blood into the cavity of the abdomen was the immediate cause of the patient's death is very evident, the author thinks, from the dissection; but how far the accident in the year 1779, or the subsequent Cæsarean section, might operate as an occasional cause, he leaves to the experienced reader to determine.

